# Bacteriophages and their potential for treatment of metabolic diseases

**DOI:** 10.1111/1753-0407.70024

**Published:** 2024-11-25

**Authors:** Youpeng Deng, Shouwei Jiang, Hanyu Duan, Haonan Shao, Yi Duan

**Affiliations:** ^1^ Department of Infectious Diseases, The First Affiliated Hospital of USTC, Division of Life Sciences and Medicine University of Science and Technology of China Hefei China; ^2^ Center for Advanced Interdisciplinary Science and Biomedicine of IHM, Division of Life Sciences and Medicine University of Science and Technology of China Hefei China; ^3^ Key Laboratory of Immune Response and Immunotherapy, Division of Life Sciences and Medicine University of Science and Technology of China Hefei China

**Keywords:** bacteriophage, gut virome, metabolic diseases, phage therapy

## Abstract

Recent advances highlight the role of gut virome, particularly phageome, in metabolic disorders such as obesity, type 2 diabetes mellitus, metabolic dysfunction‐associated fatty liver disease, and cardiovascular diseases, including hypertension, stroke, coronary heart disease, and hyperlipidemia. While alterations in gut bacteria are well‐documented, emerging evidence suggests that changes in gut viruses also contribute to these disorders. Bacteriophages, the most abundant gut viruses, influence bacterial populations through their lytic and lysogenic cycles, potentially modulating the gut ecosystem and metabolic pathways. Phage therapy, previously overshadowed by antibiotics, is experiencing renewed interest due to rising antibiotic resistance. It offers a novel approach to precisely edit the gut microbiota, with promising applications in metabolic diseases. In this review, we summarize recent discoveries about gut virome in metabolic disease patients, review preclinical and clinical studies of phage therapy on metabolic diseases as well as the breakthroughs and currently faced problems and concerns.

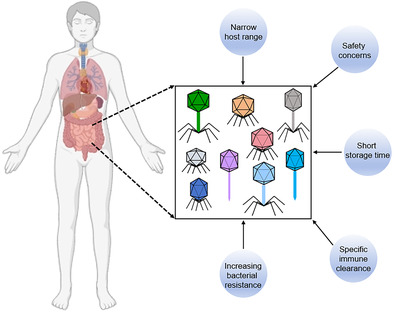

## Graphical abstract

**Highlights**

- The role of phages in preserving human health and their involvement in the pathogenesis of metabolic diseases.
- The recent advancements in phage‐based therapies, and current challenges and potential future directions.

## 1 INTRODUCTION

A wide range of human metabolic disorders, including obesity, type 2 diabetes mellitus (T2DM), metabolic dysfunction‐associated fatty liver disease (MAFLD), and cardiovascular diseases (CVDs) such as hypertension (HTN), hyperlipidemia (HLD), stroke, and coronary heart disease (CHD) have been associated with alterations in the intestinal microbiota.^1^, ^2^, ^3^, ^4^, ^5^, ^6^, ^7^, ^8^ While most of these shifts have been attributed to bacterial changes, only a few studies have revealed the particular roles of gut viruses in human diseases.^9^, ^10^, ^11^, ^12^, ^13^, ^14^, ^15^ Bacteriophages (phages), viruses that infect bacteria, are the most abundant and diverse biological entities on Earth.^16^ The human virome, largely composed of phages, plays an integral role in maintaining the gut ecosystem.^17^ Unlike other microorganisms, phages are generally classified based on their morphology, as observed through transmission electron microscopy, and their genomic sequences.^18^, ^19^ The majority of phages identified in the human gut belong to the order Caudovirales, which encompasses five families: Ackermannviridae, Herelleviridae, Myoviridae, Podoviridae, and Siphoviridae.^20^

Nonetheless, phages can further be categorized based on their life cycles as either virulent or temperate.^21^ Virulent phages, also known as lytic phages, after adhering to bacterial surface receptors and injecting their genomic DNA into the bacterial cytoplasm, can initiate a cascade of events ultimately leading to the lysis of the host cells.^22^ In contrast, lysogenic phages can pursue either a lytic or lysogenic pathway, with the decision dictated by early infection conditions. In the lysogenic cycle, genes that drive lytic cycle are silenced, and the phage genome typically integrates into the bacterial chromosome as a prophage.^23^, ^24^, ^25^, ^26^

As the natural predators of bacteria, phages often exhibit a high degree of specificity toward their bacterial hosts; their host range is usually limited to a single bacterial species or a small subset within a genus, with minimal cross‐genus infectivity.^27^ Phages were widely utilized as antimicrobial agents in Western medicine during the early twentieth century, but its prominence declined after the discovery of antibiotics, which became the predominant means of combating bacterial infections.^28^, ^29^ In recent years, however, the rise of multi‐drug resistant pathogens has reignited interest in phage therapy.^30^, ^31^ Modern phage therapy now employs phage cocktails, bioengineered phages, fecal virome transplantation, and purified endolysin derived from phages to target and destroy bacteria at infection sites.^32^, ^33^, ^34^, ^35^, ^36^, ^37^ Beyond addressing multi‐drug resistant bacterial infections, phages have demonstrated potential in precisely modulating the gut microbiota, offering new possibilities for improving the development and progression of non‐infectious diseases, including metabolic diseases.^38^, ^39^, ^40^, ^41^ Here, we examine the role of phages in preserving human health and their involvement in the pathogenesis of metabolic diseases, summarize recent advancements in phage‐based therapies, and explore current challenges and potential future directions.

## 2 INTESTINAL VIROME IN PATIENTS WITH METABOLIC DISEASES

Metabolic diseases, including obesity, type 2 diabetes mellitus, metabolic dysfunction‐associated fatty liver disease, and cardiovascular disease, represent a cluster of disorders driven by metabolic dysregulation that has evolved into a significant global health crisis.^42^, ^43^, ^44^ These conditions frequently coexist, share overlapping risk factors, and are linked to heightened risks of disability, cancer, and premature mortality.^45^ According to data from the Global Burden of Diseases, Injuries, and Risk Factors Study, there are currently 500 million cases of T2DM,^46^ 1.3 billion cases of HTN,^47^ and 1.7 billion cases of MAFLD worldwide.^48^ From 2000 to 2019, the global mortality trend for metabolic diseases consistently rose, with the highest proportional increase observed in obesity (41.09%), followed by HLD (35.98%), T2DM (12.06%), HTN (9.47%), and MAFLD (1.4%).^49^

Current therapeutic approaches for metabolic diseases are largely reliant on replacement therapies; however, these interventions often come with undesirable side effects, including weight gain, insulin resistance, and an elevated risk of cardiovascular events.^50^ Recent advances in human microbiome studies, including gut virome research, have uncovered its involvement in metabolic diseases, offering a novel and promising therapeutic avenue^51^, ^52^ (Table 1).

**TABLE 1:** Clinical studies of gut virome composition in patients with metabolic diseases.

Comparison		Outcomes	Refs.
Obesity (*n* = 10), NW (*n* = 10)	Obesity vs. NW	Increased viral richness and diversity.	59
Obesity (*n* = 396), NW (*n* = 466)	Obesity vs. NW	Decreased viral richness and diversity.	62
T2DM (*n* = 71), healthy (*n* = 74)	T2DM vs. healthy	Increased viral richness.	10
T2DM (*n* = 74), LC (*n* = 101)	T2DM vs. LC	Decreased viral richness and diversity. *Escherichia* phage, *Geobacillus* phage, and *Lactobacillus* phage were increased.	9
T2DM (*n* = 90), healthy (*n* = 42)	T2DM vs. healthy	Decreased viral richness and diversity.	64
MAFLD (*n* = 72), AUD (*n* = 62)	MAFLD vs. AUD	*Lactococcus* phages were decreased.	12
NAFLD (*n* = 73), healthy (*n* = 22)	NAFLD vs. healthy	Decreased viral richness and diversity.	68
HTN (*n* = 36), healthy (*n* = 14)	HTN vs. healthy	*Przondovirus*, *Winklervirus*, *Drulisvirus*, and *Wellingtonvirus* were increased, *Mammarenavirus* and *Oengusvirus* were decreased.	70
Stroke (*n* = 15), healthy (*n* = 15)	Stroke vs. healthy	The overall gut virome diversity was identical.	15
CHD (*n* = 37), healthy (*n* = 6)	CHD vs. healthy	Virgaviridae and Microviridae were increased.	14

Abbreviations: AUD, alcohol use disorder; CHD, coronary heart disease; HTN, hypertension; LC, lean control; MAFLD, metabolic dysfunction‐associated fatty liver disease; NAFLD, non‐alcoholic fatty liver disease; NW, normal weight; T2DM, type 2 diabetes mellitus.

### 2.1 Obesity

Obesity, defined as an excessive or abnormal accumulation of body fat detrimental to health, is a multifaceted, heterogeneous, chronic, and relapsing condition.^53^ Its complexity spans multiple dimensions, including its clinical presentation, underlying pathophysiology, progression, and response to treatment. Emerging evidence suggests a causal relationship between microbial dysbiosis and obesity.^54^ For instance, numerous studies have confirmed a reduction in gut microbial diversity and alterations in microbiota composition in individuals with obesity compared with those with normal weight.^1^, ^55^, ^56^, ^57^ However, these investigations predominantly focus on bacterial changes in obesity, often neglecting the role of the gut virome. Recent studies have demonstrated that phages, particularly those belonging to Caudovirales, dominate the gut microbiota.^58^ Some researches have also identified an increased richness of certain phage contigs linked to the transition from normal weight to obesity, with several phage contigs significantly overrepresented in individuals with obesity.^59^, ^60^ In a preclinical study, Kim et al. conducted a metagenomic analysis to investigate the composition of the mucosal and luminal virome in the gut and assessed the impact of a Western diet on gut viral ecology. They found that mucosal and luminal viral assemblages predominantly consisted of temperate phages with the hosts from Bacilli, Negativicutes, and Bacteroidia classes, with a marked enrichment of temperate phages in the gut virome of obese mice. These changes were more pronounced in the mucosa than in the lumen, leading to a loss of spatial differentiation, though they were reversed upon switching to a low‐fat diet.^61^ Moreover, a meta‐analysis of metagenomic sequencing data from obese and non‐obese individuals revealed a significant decrease in both the richness and diversity of the gut bacteriome and virome in obese patients. Five viral families, including Mesyanzhinovviridae, Chaseviridae, Salasmaviridae, Drexlerviridae, and Casjensviridae, showed altered abundance. Gut viral operational taxonomic units exhibited a diagnostic accuracy with an optimal area under the curve of 0.766 in distinguishing obesity from healthy controls.^62^

### 2.2 Diabetes mellitus

Diabetes mellitus (DM), closely linked to obesity, encompasses a group of metabolic disorders characterized by chronic hyperglycemia.^45^ The World Health Organization classifies DM into two major types: type 1 DM (T1DM) and type 2 DM (T2DM). T2DM is the most prevalent chronic metabolic disorder and has become an increasing public health concern, particularly in Western countries.^63^ In recent years, studies have suggested that the gut virome plays a critical role in the progression of T2DM, though the majority of research has traditionally concentrated on gut bacteria. For instance, Ma et al. utilized a whole‐community metagenomic sequencing dataset derived from fecal samples from 370 Chinese T2DM patients and non‐diabetic controls and employed three bioinformatic strategies to identify large scaffolds of phage origin and define phage operational taxonomic units to explore the human gut phageome.^10^ This was the first study to associate the gut phageome with T2DM, though it did not elucidate the specific mechanisms by which the phageome contributes to T2DM risk. Another study revealed that obese subjects with T2DM (ObT2) exhibited reduced gut viral richness and diversity compared with lean controls. Seventeen viral species were linked to ObT2 individuals compared with lean controls, with four viral group (*Micromonas pusilla* virus, *Cellulophaga* phage, *Bacteroides* phage, and *Halovirus*) increased in ObT2, while 13 groups, including *Hokovirus*, *Klosneuvirus*, and *Catovirus*, were decreased.^9^ Additionally, a recent study containing 132 participants found that Caudovirales were the predominant gut viruses, with six viral taxa significantly decreased in T2DM patients compared with healthy controls at the order level. At the family level, Microviridae were the dominant viruses, with 12 viral families showing significant differences between groups. The study also identified disrupted viral–bacterial interactions in T2DM patients compared with healthy subjects.^64^ Notably, similar reductions in alpha diversity and alterations in viral composition have been observed in other metabolic diseases, suggesting that the gut virome may play a critical role in their development.

### 2.3 Metabolic dysfunction‐associated fatty liver disease

Metabolic dysfunction‐associated fatty liver disease, formerly called non‐alcoholic fatty liver disease (NAFLD), encompasses a spectrum of conditions ranging from simple hepatic steatosis to non‐alcoholic steatohepatitis, fibrosis, cirrhosis, and hepatocellular carcinoma.^65^, ^66^ Within MAFLD, patients with a higher MAFLD activity score (MAS) and greater degrees of liver fibrosis on histology are at higher risk for disease progression, hepatocellular carcinoma, and liver‐related mortality.^67^ A prospective, cross‐sectional observational study found that patients with MAFLD and MAS 5–8 had significantly lower intestinal viral diversity compared with patients with MAS 0–4 or control individuals. Advanced MAFLD was associated with a marked reduction in the proportion of phages relative to other intestinal viruses.^68^ This landmark study links histologic markers of MAFLD severity to significant decreases in viral diversity and phage proportions, developing a model that identifies patients with severe MAFLD and fibrosis more accurately than models based solely on clinical or bacterial data. Furthermore, a recent study noted substantial differences in the intestinal virome of MAFLD and alcohol use disorder (AUD) patients. The relative abundance of certain *Lactococcus* phages was lower in MAFLD patients compared with AUD patients, and multivariate modeling using the most distinguishing *Lactococcus* phages more effectively predicted alcohol use in the MAFLD population than the AUD/MAFLD Index.^12^

### 2.4 Cardiovascular disease

Cardiovascular diseases, including hypertension, stroke, and coronary heart disease, are associated with high morbidity and mortality, making them the leading cause of death worldwide.^69^ A recent study found that four viral genera (*Przondovirus*, *Winklervirus*, *Drulisvirus*, and *Wellingtonvirus*) were increased, while two viral genera (*Mammarenavirus* and *Oengusvirus*) were decreased in HTN patients compared with healthy controls, though no significant changes were observed at the family level.^70^ In another study, Han et al. speculated that alterations in gut viral composition, particularly the abundance of *Erwinia* phage phiEaH2 and *Lactococcus* phage 1706, may drive changes in viral types and could be linked to the development of hypertension.^13^ Stroke, another major CVD with high rates of mortality and disability, has also been associated with changes in the gut virome. Wang et al. reported that while overall gut virome diversity did not differ significantly between stroke patients and healthy controls, virome composition varied substantially, with the relative abundance of *Bacteroides* phage B40_8 and *Cronobacter* phage CS01 significantly elevated in stroke patients.^15^ Additionally, CHD, the second leading cause of cardiovascular mortality in China, is also related to the gut virome composition.^71^ A recent study suggested that virome composition in CHD patients is linked to dietary habits and medical therapy. Virgaviridae (69.48%) and Microviridae (21.05%) were the dominant viral families in the gut virome of CHD patients, with Virgaviridae, a family of rod‐shaped plant viruses, likely reflecting the patients' increased consumption of plant‐based foods to reduce body fat and improve health.^14^ Collectively, accumulating evidence underscores the significance of gut viruses in CVDs, but further research is necessary to fully elucidate the interactions between host, viruses, and bacteria to better understand the biological impact of manipulating the gut virome.

## 3 PHAGE‐BASED THERAPY IN METABOLIC DISEASES

The first instance of phage therapy was documented in 1919 when d'Hérelle and his colleagues successfully used phages to treat chickens infected with *Salmonella gallinarum*.^72^ Following this, d'Hérelle employed phage therapy in humans, observing that introducing an anti‐cholera phage into the drinking wells of villages during an outbreak effectively prevented further infections.^73^ However, with the advent of antibiotics, phage therapy gradually fell into obscurity.^74^ Today, due to the growing threat of antibiotic‐resistant bacterial pathogens, phage therapy is experiencing a resurgence. Phages have the ability to selectively and efficiently target and kill antibiotic‐resistant bacteria at the end of their infection cycle.^75^ For instance, a systematic review on the safety and efficacy of phage therapy for superficial bacterial infections found that purified phages can effectively treat antibiotic‐resistant bacterial infections through various administration routes, with no adverse effects reported.^76^ Additionally, recent studies have explored the use of phage therapy to reshape gut microbiota and treat metabolic diseases. Duan et al. demonstrated that phages could specifically target cytolytic *Entercoccus faecalis*, a bacterium associated with the severity of liver disease and mortality in alcoholic hepatitis patients, offering a method for the treatment of alcohol‐associated liver disease by precise editing of the intestinal microbiota.^38^ In another study, Gan and colleagues identified that high alcohol‐producing *Klebsiella pneumoniae* (HiAlc *Kpn*) in the gut microbiome may contribute to NAFLD. They utilized *Klebsiella*‐specific phages to treat mice gavaged with HiAlc *Kpn*, finding that phage therapy alleviated HiAlc *Kpn*‐induced steatohepatitis, including hepatic dysfunction and the expression of cytokines and lipogenic genes.^40^ Similarly, Ye et al. found that administering a phage cocktail significantly altered the gut microbiome, ameliorating the dysbiosis observed in diabetic mice induced by high‐fat diet and streptozotocin. This was achieved by influencing microbial composition as well as metabolic pathways and metabolites.^77^

Fecal microbiota transplantation (FMT) has been proven to be an effective therapy for gut dysbiosis‐related diseases, as it can restore the composition and function of imbalanced gut microbiota.^78^ With the advancements in next‐generation sequencing (NGS), numerous studies have highlighted the close relationship between the gut virome and diseases, pointing out the key roles of virome in shaping gut microbiota composition and influencing the host metabolome.^79^, ^80^ Consequently, fecal virome transplantation (FVT) has emerged as a promising refinement of FMT, offering improved methodologies. A murine study showed that autochthonous virome transfer could reshape the microbiome of mice after antibiotic treatment, bringing them closer to the pre‐antibiotic microbiota profile compared with those receiving non‐viable phages.^81^ Another study demonstrated that FVT from lean phenotype mice to obese phenotype mice reduced weight gain, normalized blood glucose levels, and enhanced the expression of genes involved in energy homeostasis. Borin et al. also confirmed that FVT could alter the fecal microbiome in animal model, showing that it can drive lean and obese phenotypes in mice.^82^ In a recent double‐blind, randomized, placebo‐controlled clinical trial, Wortelboer et al. provided proof of concept that FVT from lean healthy donors containing gut virions can be safely administered to recipients with metabolic syndrome. The study demonstrated that gut phages have the potential to improve glycemic variability and modulate phage‐bacteria dynamics.^83^

## 4 CHALLENGES OF PHAGE THERAPY

**FIGURE 1:**
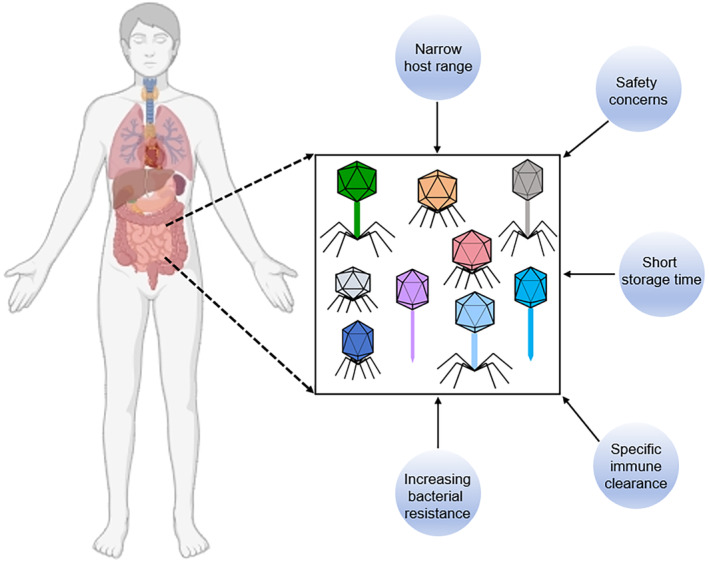
Challenges that hinder the phage therapy. As the issue of antibiotic resistance currently becomes ever more pressing, phage therapy presents significant potential for therapeutic applications. However, it also confronts numerous challenges, including safety concerns, narrow host range, specific‐phage immune clearance, increasing bacterial resistance, and short storage time.

With the rise of antibiotic‐resistant bacteria, phages have emerged as promising alternatives to antibiotics due to their specificity in targeting pathogens and their ability to kill them efficiently. However, despite their potential, several challenges hinder the widespread adoption of phage therapy in clinical practice (Figure 1).

One of the primary concerns about phage therapy is its safety.^84^, ^85^ However, substantial preclinical and clinical studies have demonstrated that phage therapy is generally safe and well‐tolerated in most cases.^86^, ^87^, ^88^, ^89^ In a preclinical study, Dufour et al. showed that phage 536_P1 effectively eradicated *Escherichia coli* infection without eliciting an innate inflammatory response.^90^ Additionally, Drilling et al. demonstrated that treating *Staphylococcus aureus*‐associated rhinosinusitis in sheep with the phage cocktail NOV012 over 20 days was safe, with no observed inflammatory infiltration or tissue damage in the sinus mucosa.^91^ In a double‐blind, placebo‐controlled crossover trial, Gindin et al. demonstrated that administering therapeutic doses of a blend of four phages was both safe and well‐tolerated within the target human population.^92^ Similarly, in another randomized, placebo‐controlled, double‐blind clinical trial, Leitner et al. observed that intravesical phage therapy was non‐inferior to standard antibiotic treatment, with a favorable safety profile for the phage therapy.^93^ Recently, a couple case reports also suggested that phage therapy is safe. Johri et al. utilized phages to treat a patient with chronic bacterial prostatitis, revealing that phage therapy held the potential for infection control and inflammation reduction without adverse reactions.^94^ Furthermore, another clinical case study involving a 61‐year‐old woman being successfully treated after a second cycle of phage therapy underscored the safety and efficacy of both intravenous and intra‐articular infusions. This successful outcome, achieved with a single lytic phage, also highlighted the development of serum neutralization with prolonged treatment.^95^ Nevertheless, some studies have reported transient adverse events or side effects during phage therapy, including inflammation, flushing, hypotension, and fever.^96^, ^97^ Thus, while recent successes in preclinical and clinical studies have illustrated that phages generally possess a favorable safety profile and are typically well‐tolerated in both animal and human subjects, it remains critical to continue expanding our knowledge base and advancing the translatability of phages from bench‐side research to clinical applications.

Another challenge is the limited host range of phages. While their specificity can be advantageous by sparing commensal microbiota, it complicates the rapid selection of an appropriate phage to combat infections.^98^, ^99^ There are three common strategies to address this issue. Combining phages with different host ranges in a single cocktail is the most common approach to broaden the target spectrum.^100^ Studies have shown that phage cocktails are more effective at reducing bacterial mutation frequency and rescuing murine bacteremia than single‐phage treatments.^101^ Recently, genetic engineering techniques have been used to modify or expand the host range of phages, typically by altering their tail fibers.^102^ For example, Marzari et al. expanded the host range of the filamentous coliphage fd by adding a receptor‐binding domain from phage IKe, enabling it to infect *E. coli* strains with N‐pili.^103^ Similarly, Yosef et al. designed hybrid particles with various phage tail proteins, enhancing DNA transfer into new hosts.^104^ Besides, phage adaptation, a process of selecting evolved phages with broader host ranges in the laboratory, has also been used.^105^, ^106^ Friman and colleagues demonstrated that evolved phages were more effective at reducing bacterial densities than ancestral phages, partly because fewer bacterial strains could evolve resistance to evolved phages.^106^ In conclusion, while these methods represent the most commonly employed strategies for broadening the host range of phages, each approach carries inherent limitations in clinical application. For instance, phage cocktails, though frequently utilized in clinical practice, may fail to target all bacterial strains within a species, and the varied composition of the cocktail might unintentionally affect non‐target bacteria. Thus, there remains a pressing need to devise additional strategies to broaden the host range of phages.

Bacterial resistance to phages is another significant challenge, as bacteria can thwart phage attacks through antiviral mechanisms.^107^ Some studies have shown that bacteria can rapidly develop resistance to phages, leading to uncontrolled proliferation of phage‐resistant mutants and treatment failure.^108^ Among 12 phage therapy clinical studies, seven confirmed the emergence of phage resistance during treatment.^109^ However, phage resistance does not always result in treatment failure, as new phages can be selected, and the patient's immune system may eradicate resistant bacterial mutants.^110^, ^111^

Phages, as foreign substances, can stimulate phage‐specific immune responses, potentially compromising their activity and reducing their efficacy.^112^, ^113^ Some studies have suggested that phages can trigger the generation of specific antiviral antibodies, which can neutralize phages by binding to their tails and affect their antibacterial activity.^114^, ^115^, ^116^ Recent studies have shown that neutralizing immune responses are dose‐dependent, with weaker or ineffective immune responses observed at lower phage doses.^117^, ^118^ However, determining optimal low‐dose administration is challenging due to various uncontrollable factors, and no universal route of phage administration currently exists to ensure efficient delivery and effective dosing. To address these issues, researchers have explored encapsulating phages, as phages within liposomes have demonstrated advantageous therapeutic properties over free phage administration.^119^

The formulation of phages for long‐term storage and preservation is another significant obstacle to their industrialization and clinical application, as phage capsids are proteins susceptible to denaturation. In 1962, Clark found that phages stored at 4°C had better titers than those kept at room temperature, though titers declined over time regardless of storage conditions.^120^ Currently, lyophilization (freeze‐drying) is the most common preservation method for protein‐based compounds. Studies have shown that lyophilized phages remain stable at −80°C for up to 10 years, though their activity declines by 1 log_10_ per year.^121^ In addition to lyophilization, spray drying is another method, which must be kept below 40°C to avoid denaturation and inactivation of phages. Leung et al. compared spray freeze drying (SFD) and spray drying (SD) methods for producing inhalable phage powders, finding that the SD method caused less phage reduction during the process and produced powders with better aerosol performance for a *Pseudomonas* phage.^122^

## 5 CONCLUSIONS

Phage therapy has proven to be a powerful tool for treating bacterial infections for over a century, especially in the context of rising antibiotic resistance. Beyond its antibacterial applications, recent reports suggest that phage therapy holds significant promise as an emerging therapeutic approach for human metabolic diseases, such as obesity, T2D, HTN, HLD, MAFLD, and CVDs. Currently, phage therapy for metabolic diseases is expected to develop into a pre‐defined phage‐based medicinal product. However, numerous unknowns remain, and much research is needed before its clinical application.

## AUTHOR CONTRIBUTIONS

Youpeng Deng and Shouwei Jiang wrote the original manuscript; Hanyu Duan and Haonan Shao provided assistance in writing the manuscript; Shouwei Jiang and Yi Duan were responsible for project administration, conceptualization, supervision and manuscript editing.

## CONFLICT OF INTEREST STATEMENT

The authors declare no conflict of interest.
